# Identification of Genes that Maintain Behavioral and Structural Plasticity during Sleep Loss

**DOI:** 10.3389/fncir.2017.00079

**Published:** 2017-10-23

**Authors:** Laurent Seugnet, Stephane Dissel, Matthew Thimgan, Lijuan Cao, Paul J. Shaw

**Affiliations:** ^1^Centre de Recherche en Neurosciences de Lyon, U1028/UMR 5292, Team WAKING, Université Claude Bernard Lyon 1, INSERM U1028, CNRS UMR 5292, Lyon, France; ^2^Department of Neuroscience, Washington University School of Medicine, St. Louis, MO, United States; ^3^Department of Biological Sciences, Missouri University of Science and Technology, Rolla, MO, United States

**Keywords:** sleep, plasticity, learning, memory, homeostasis, *Drosophila*, ubiquitin, GABA-A receptors

## Abstract

Although patients with primary insomnia experience sleep disruption, they are able to maintain normal performance on a variety of cognitive tasks. This observation suggests that insomnia may be a condition where predisposing factors simultaneously increase the risk for insomnia and also mitigate against the deleterious consequences of waking. To gain insight into processes that might regulate sleep and buffer neuronal circuits during sleep loss, we manipulated three genes, *fat facet* (*faf)*, *highwire* (*hiw*) and the GABA receptor *Resistance to dieldrin* (*Rdl*), that were differentially modulated in a *Drosophila* model of insomnia. Our results indicate that increasing *faf* and decreasing *hiw* or *Rdl* within wake-promoting large ventral lateral clock neurons (lLNvs) induces sleep loss. As expected, sleep loss induced by decreasing *hiw* in the lLNvs results in deficits in short-term memory and increases of synaptic growth. However, sleep loss induced by knocking down *Rdl* in the lLNvs protects flies from sleep-loss induced deficits in short-term memory and increases in synaptic markers. Surprisingly, decreasing *hiw* and *Rdl* within the Mushroom Bodies (MBs) protects against the negative effects of sleep deprivation (SD) as indicated by the absence of a subsequent homeostatic response, or deficits in short-term memory. Together these results indicate that specific genes are able to disrupt sleep and protect against the negative consequences of waking in a circuit dependent manner.

## Introduction

In humans, sleep disruption has debilitating consequences on daytime functioning and health (Banks and Dinges, 2007). As a consequence, insomnia has an important economic burden with both direct (medical) and indirect (e.g., absenteeism, accidents) costs (Ozminkowski et al., 2007). Unfortunately the neuronal mechanisms underlying insomnia and their relationship to cognitive deficits are poorly understood. For example, patients with primary insomnia experience sleep disruption, for 4 years or more (Chevalier et al., 1999) and while substantially impaired (Hauri, 1997; Drake et al., 2003; Buysse et al., 2007; Edinger et al., 2008) are able to maintain normal performance on a variety of tasks (Riedel and Lichstein, 2000; Orff et al., 2007). The ability to maintain normal performance is surprising given the well-established negative impact of inadequate sleep on a variety of cognitive tests (Krause et al., 2017).These observations suggest that insomnia may be a condition where predisposing factors simultaneously increase the risk for insomnia and also mitigate against the deleterious consequences of waking. Understanding how patients with insomnia can maintain cognitive abilities during sleep loss may provide novel insights into the relationship between sleep and plasticity.

Using a laboratory selection strategy, we have isolated a population of flies (*insomniac-like* flies referred to as *ins-l* flies) that share many features with human insomnia, including difficulty falling asleep, difficulty staying asleep, inadequate sleep duration and poor quality sleep as evidenced by daytime cognitive impairments (Seugnet et al., 2009c). Whole genome profiling identified a large set of genes (~1000) that were differentially regulated in *ins-l* flies (Seugnet et al., 2009c). Among the genes differentially expressed in *ins-l* flies, 30 genes are involved in synaptic transmission and constituted a significantly over-represented biological process as defined by Gene Ontology. These genes represent a possible link between synaptic activity, sleep regulation and behavioral performance. Indeed, two of these genes, *highwire (hiw)* an E3 ubiquitin ligase (DiAntonio and Hicke, 2004), and *Resistant to dieldrin* (*Rdl*), a GABA-A receptor (Henderson et al., 1993), enhance memory under some conditions (Liu et al., 2007; Huang et al., 2012). Given their role in both memory, and sleep (see below) the ubiquitin-proteasome system and GABA signaling represent two pathways that are uniquely situated to not only modulate sleep time but also buffer neuronal circuits during sleep loss.

The ubiquitin-proteasome system plays an important role in activity- dependent plasticity and has recently been implicated in regulating sleep (Stavropoulos and Young, 2011; Freeman et al., 2012; Pfeiffenberger and Allada, 2012; Jarome and Helmstetter, 2013). Specifically, flies mutant for an adaptor for the *Cullin-3* ubiquitin ligase complex*, insomniac*, display dramatic reductions in total sleep time (~400 min/day). Moreover, total sleep is also reduced when either *insomniac* (*inc*) or *Cullin-3* (*Cul3*) are knocked down using RNAi. While these data strongly implicate the ubiquitin-proteasome complex as regulators of sleep time, changes in sleep were only observed when *inc* and *Cul-3* were knocked down pan-neuronally throughout development but did not reduce sleep when they were manipulated in adults (Pfeiffenberger and Allada, 2012). In comparison, data from human patients with insomnia indicate that distinct neuronal circuits contribute to various insomnia phenotypes in adults (Nofzinger et al., 2004). Thus, a closer evaluation of the circuits impacted by the ubiquitin-proteasome system is warranted.

GABA signaling has also been shown to modulate sleep in flies (Agosto et al., 2008; Parisky et al., 2008; Chung et al., 2009). The role of the GABA-A receptor *Rdl* in regulating sleep onset was first identified by demonstrating that the wake-promoting effects of the anticonvulsant, carbamazepine, were reduced in *Rdl* mutants (Agosto et al., 2008). Based upon the immunohistochemical localization of *Rdl*, genetic studies localized the sleep-regulating properties of *Rdl* to a subset of clock neurons, the ventral lateral neurons (LNvs; Parisky et al., 2008; Chung et al., 2009). Although subsequent studies have begun to investigate molecular mechanisms regulating the *Rdl* receptor in the LNvs (Li et al., 2013; Liu et al., 2014), little is known about how modulating the *Rdl* receptor in the LNvs, or other brain regions, impacts behavioral plasticity.

As mentioned above, insomnia patients perform well on a variety of cognitive tasks despite experiencing inadequate sleep (Riedel and Lichstein, 2000). Of the genes differentially expressed in insomnia-like flies, the only genes that both disrupt sleep and enhance memory are those involved in the ubiquitin-proteasome system (*hiw*) and *Rdl*. Although *hiw* and *Rdl* do not appear to be functionally related, human studies have identified a number of unrelated genes that are associated with insomnia phenotypes (for review see Lind and Gehrman, 2016). Moreover, whether distinct genes influence insomnia is predicted to be differentially influenced by changes in the environment (Lind and Gehrman, 2016). That is, the literature predicts that genes with independent function will likely influence insomnia phenotypes in different ways. With that in mind, we asked whether *hiw* and *Rdl* could independently influence sleep and cognitive impairments during sleep loss. Surprisingly, our results indicate that the ability of *faf*, *hiw* or *Rdl* to modulate sleep and protect against cognitive impairments during waking is circuit dependent. These data provide additional insight into potential mechanisms that allow patients with insomnia to maintain cognitive ability during sleep disruption.

## Materials and Methods

### Fly Stocks, Sleep Monitoring and Sleep Deprivation

*Cs* flies were obtained from the Bloomington *Drosophila* stock center. *UAS-hiw^RNAi^* lines were obtained from the Vienna *Drosophila* RNAi center (VDRC; Dietzl et al., 2007). We obtained *faf^EP3520^*, *UAS-hiw^∆RING^* (UAS-*hiw^DN^*)stocks from A. DiAntonio (Washington University in St.Louis, MO, USA), *UAS-hiw^RNAi^* from the Vienna Drosophila Resource Center. GAL4 lines were selected based upon their expression throughout the brain or in neuronal populations known to be involved in sleep and plasticity. Drivers expressing broadly throughout the brain include elav-GAL4, MJ85B-GAL4, Cha-GAL4 and TH-GAL4. The Mushroom Bodies (MBs) play a role in sleep regulation and memory; drivers that express in the MBs include 247-Gal4, 30Y-GAL4, c309-GAL4, and 201y. The central complex drivers have been implicated in plasticity and include c232-GAL4 and c205-GAL4. The pars intercerebralis drivers include 50y-GAL4, c687-GAL4, Jan191-GAL4 and Feb194-Gal4. Finally, we obtained drivers expressing the circadian clock including *cry39-GAL4*, *cry16-GAL4, cry-Gal80* stocks from M. Rosbash (Brandeis University), *PDF-GAL4* flies from P. Taghert (Washington University in St.Louis, MO, USA). *UAS-Rdl RNAi* stocks and other GAL4 drivers were obtained from Bloomington stock center, P. Taghert, A. DiAntonio, S. Birman (Université de la Mediterannée), R. Greenspan (Neuroscience Institute, San Diego, CA, USA), A. Sehgal (University of Pennsylvania), and R. Allada (Northwestern University). For GAL4:UAS experiments, parental lines were outcrossed to *Cs* flies. Flies were cultured at 25°C, 50% to 60% humidity, in 12 h:12 h Light:Dark cycle, on a standard food containing yeast, dark corn syrup, molasses, dextrose and agar. Newly eclosed female adult flies were collected from culture vials daily under CO_2_ anesthesia. Three day old flies were then individually placed into 65 mm glass tubes so that sleep parameters can be continuously evaluated using the Trikinetics activity monitoring system as previously described (Shaw et al., 2000)^1^. Flies were sleep deprived using an automated SD apparatus that has been found to produce waking without nonspecifically activating stress responses (Shaw et al., 2002). Flies were sleep deprived using the SNAP from ZT 12 (beginning of the dark phase) to ZT 0 (beginning of the light phase). Unless otherwise stated, at least 16 flies were analyzed for each experimental condition. Differences in sleep time were assessed using either a Student’s *t*-test or analysis of variance (ANOVA) which were followed by planned comparisons using a Modified Bonferroni correction.

### Locomotor Rhythms

Flies were individually placed into 5 × 65 mm tubes with regular food and placed into constant conditions for 10 days. Locomotor activity was continuously recorded in 30 min bins using the Trikinetics system. Locomotor rhythms were analyzed for 6 or 9 days using MATLAB (Mathworks, Natick, MA, USA) based computational tools designed by Levine et al. (2002). At least 30 flies were analyzed for each genotype.

### Learning

The APS performance test was performed as previously described (Le Bourg and Buecher, 2002). Flies are individually tested in a T-maze where they are allowed to choose between a dark and a lighted vial. Adult flies are phototaxic and choose the lighted alley in the absence of reinforcer. During the test a filter paper soaked with a quinine solution is placed in the lighted vial to provide an aversive association. In the course of 16 trials through the maze flies learn to make more frequent choices to the dark vial (photonegative choices). The number of photonegative choices is tabulated during four successive blocks of four trials and the performance score is the % of photonegative choices made in the last block of four trials. At least eight flies were evaluated for each condition. For each experiment, learning was evaluated by the same experimenter who was blind to genotype and condition. All flies were tested in the morning between ZT0 and ZT4. Flies were sleep deprived using the SNAP from ZT 12 (beginning of the dark phase) to ZT 0 (beginning of the light phase) and until each flies was tested for learning. Learning scores are normally distributed (Seugnet et al., 2009b). Differences between scores were assessed using either a Student’s *t*-test or ANOVA which were followed by planned comparisons using a Modified Bonferroni correction.

### Photosensitivity

Photosensitivity was evaluated using the T-maze with no filter paper. The average proportion of choices to the lighted vial during 10 trials was calculated for each individual fly. The phototaxis index (PI) is the average of the scores obtained for at least 5 flies ± SEM.

### Quinine/Humidity Sensitivity

Sensitivity to quinine/humidity was evaluated as Le Bourg and Buecher (2002) with the following modifications: each fly was individually placed in a 14 cm transparent cylindrical tube covered with filter paper, uniformly lighted and maintained horizontal. In one half of the apparatus the filter paper is soaked with quinine solution while the other half is kept dry. The quinine/humidity sensitivity index (referred to as Quinine Sensitivity Index or QSI) was determined by calculating the time in seconds that the fly spent on the dry side of the tube when the other side had been wetted with quinine, during a 5 min period.

### Immunohistochemistry

Brains were removed from the head casing and fixed in 4% paraformaldehyde in phosphate buffer solution (PBS; 1.86 mM NaHPO, 8.41 mM NaHPO, and 175 mM NaCl) for 1 h and washed in PBS. Following a 2-h pre-incubation in 3% normal goat serum in PBS-TX (PBS containing 0.3% Triton X-100), brains were washed in PBS-TX. Primary antibodies were Rabbit anti-GFP (1:1000; Sigma) and mouse anti-PDF (1:50; DSHB, University of Iowa), washed in PBS-TX and incubated in the appropriate fluorescent secondary antibodies.

### Confocal Microscopy

Confocal images with a 1 μm slice thickness were collected on an Olympus microscope provided by the Washington University Center for Cellular Imaging in St. Louis, MO, USA. Confocal stacks of PDF terminals were quantified as described previously (Donlea et al., 2009). Briefly, immuno-positive terminals were counted using the ImageJ binary thresholding algorithm. The number of synaptic terminals for the GAL4/+ parental control used to generate a mean. The mean of the GAL4/+ was used to normalize each individual UAS/+ and GAL4>UAS brain. The individual normalized values were then used to calculate the mean and standard error for the group. The normalized values for each group were then evaluated using a one-way ANOVA and a modified Bonferroni test.

### Statistics

All comparison were done using a Student’s *T*-test or, if appropriate, ANOVA and subsequent modified Bonferroni comparisons unless otherwise stated. All statistically different groups are defined as *p* < 0.05.

## Results

### Circuit-Dependent Regulation of Sleep by *faf* and *hiw*

**Figure 1:**
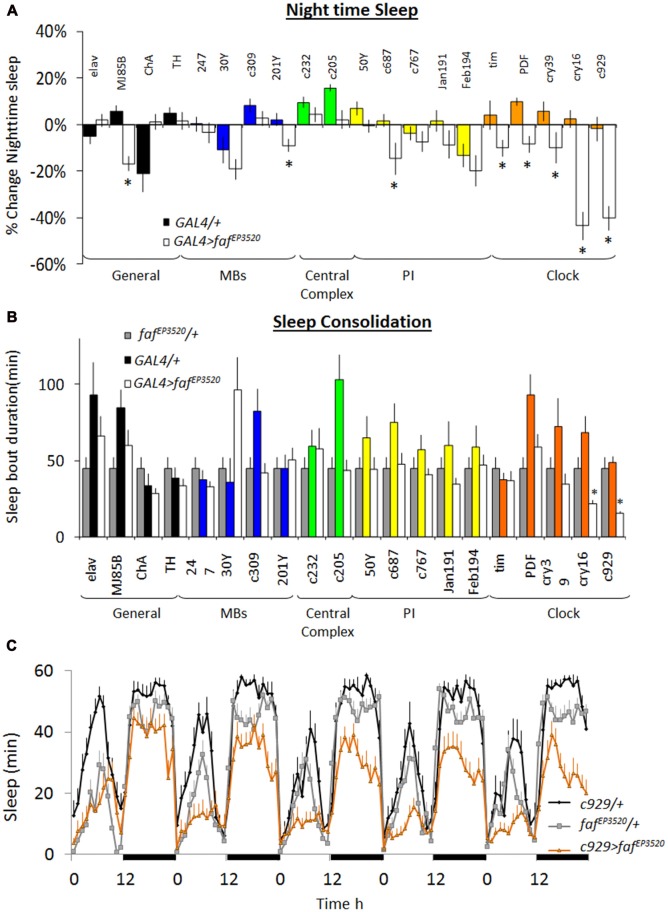
Over-expression of fat facet (faf) modulates sleep in a circuit dependent manner. **(A)** Change in night-time sleep following *faf* over-expression with GAL4 drivers that target different areas of the brain. Nighttime sleep is expressed as %change from *faf^EP3520^*/+ at day 8; (*n* > 14/group; *p < 0.05). **(B)** Average night bout duration at day 8. Gray bars: *faf^EP3520^*/+; colored bars: *GAL4/+*; white bars: *GAL4>faf^EP3520^* combinations (*n* > 14/group; *p < 0.05). **(C)** Sleep is reduced in *c929-GAL4/+; faf^EP3520^*/+ flies compared to parental controls. Data is presented as minutes of sleep/h starting on day 3; black bars indicate lights-off (one of three replicates of *n* > 16/group).

As mentioned, whole genome profiling revealed that *faf* was increased in *ins-l* flies compared to normal sleeping background controls identifying it as a potential contributor to insomnia phenotypes (Seugnet et al., 2009c). The increase of *faf* in *ins-l* flies was confirmed in an independent cohort using QPCR (data not shown). Given this expression profile, we hypothesized that over-expressing *faf* would recapitulate important features of the insomnia phenotype such as reduced sleep time and sleep fragmentation. The P-element line, *faf^EP3520^*, contains a UAS that can be used to induce functional *faf* (DiAntonio et al., 2001). Thus, we conducted a mini-brain screen to express *faf* in circuits known to modulate sleep and waking in flies (Joiner et al., 2006; Pitman et al., 2006; Foltenyi et al., 2007; Sheeba et al., 2008; Donlea et al., 2011). Although previous reports demonstrate that knocking-down *inc* and *Cul-3* with the pan-neuronal *elav-GAL4* driver substantially reduced sleep time (Stavropoulos and Young, 2011; Pfeiffenberger and Allada, 2012), *elav-GAL4>faf^EP3520^* did not reduce night-time sleep compared to genetic controls (Figure 1A, left). However, we observed a ~20% reduction in total sleep time when we expressed *faf^EP3520^* using *MJ85b-GAL4*, a stronger driver that expresses broadly throughout the brain (Joiner and Griffith, 1997; Dubnau et al., 2001); no changes in sleep were observed when *faf* was expressed in cholinergic or dopaminergic neurons (Figure 1A). Surprisingly, expressing *faf* in other sleep/wake centers (e.g., Mushroom Bodies (MBs), Fan Shaped body (FB), Central Complex, Pars Intercerebralis (PI)) did not consistently alter sleep parameters (Figure 1A). In contrast, all five GAL4 drivers that express in clock neurons significantly reduced night-time sleep when compared to both parental controls. Indeed, the only drivers that reduced night-time sleep and also disrupted sleep consolidation when expressing *faf^EP3520^* were *cry16-GAL4* and *c929-GAL4* (Figures 1A,B). Since insomnia tends to become more prevalent with age in humans, we asked whether expressing *faf* in clock cells would disrupt sleep from the first day of life, similar to the results seen with *inc* and *Cul-3*, or whether the changes in sleep would develop over time in adults. In wild-type flies, sleep reaches a stable level by 3-days of age (Shaw et al., 2000). We did not observe differences in sleep in young *c929-GAL4>faf^EP3520^* and their parental controls during their first 2 days of adult life (data not shown). Similarly, *c929-GAL4/+* (black), *faf^EP3520^*/+ (gray) and *c929-GAL4>faf^EP3520^* (orange) flies slept similarly on day 3 (Figure 1C, left). However, while sleep remained stable over days in *c929-GAL4/+* and *faf^EP3520^*/+ controls, sleep progressively declined in mature *c929-GAL4>faf^EP3520^* flies and stabilized by 7–8 days of age (Figure 1C). Thus, disrupting the ubiquitin-proteasome system in clock cells (see below) by expressing a gain-of-function allele of *faf* disrupts sleep in mature adults, and recapitulates two key features seen in *ins-l* flies.

**Figure 2:**
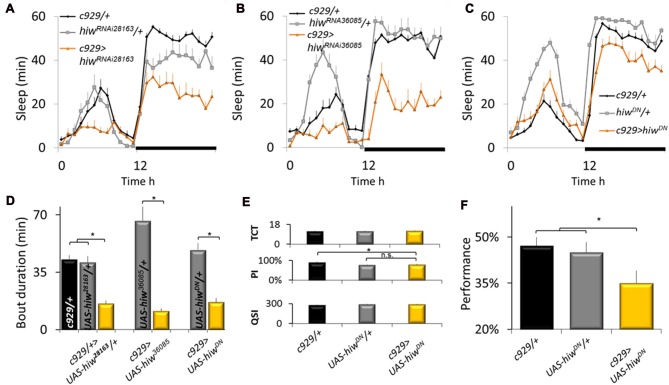
Disrupting highwire (hiw) increases waking. **(A–C)** Reducing *hiw* in c929 expressing cells using *hiw^RNAi28163^*, *hiw^RNA36085^*, or *UAS-hiw^DN^* disrupts sleep. A Genotype (3) X Time (24) repeated measures analysis of variance (ANOVA) revealed a significant Genotype × Time interaction, ANOVA *F*_(46,11058)_ = 4.96; *p* = 9.9E-16, *F*_(46,1196)_ = 5.97; *p* = 9.9E-16, and *F*_(46,996)_ = 5.26; *p* = 9.9E-16, respectively. **(D)** Disrupting *hiw* reduces average sleep bout duration at night, one-way ANOVA for Genotype *F*_(6,93)_ = 6.58, *p* = 7.97E-006; *p < 0.05 Modified Bonferroni test. **(E)** *c929-GAL4*>*UAS-hiw^DN^* flies exhibit normal values for the Time to complete Test (TCT), the Photosensitivity Index (PSI) and the Quinine Sensitivity Index (QSI) that are not statistically different from both *c929-GAL4/+* and *UAS-hiw^DN^*/+ parental controls, one-way ANOVA *F*_(2,26)_ = 0.22, *p* = 0.8, ANOVA *F*_(2,14)_ = 6.3, *p* = 0.01, ANOVA *F*_(2,14)_ = 0.73, *p* = 0.9, respectively; *p < 0.05 modified Bonferroni test). **(F)** Baseline learning is reduced in flies expressing *UAS-hiw^DN^* in c929 expressing cells one-way ANOVA *F*_(2,26)_ = 3.54, *p* = 0.042; *p < 0.05 modified Bonferroni test).

To further evaluate the role of ubiquitination pathways for sleep regulation, we obtained loss-of-function alleles for the E3 ubiquitin ligase *highwire* (*hiw*). As mentioned above, loss-of-function alleles for *hiw* phenocopy gain-of-function alleles for *faf*. As seen in Figures 2A,B knockdown of *hiw* in *c929-GAL4* expressing neurons reduced sleep time using two independent RNAi lines that target a different portion of the gene. Reduced sleep was also observed when expressing a dominant negative form of *highwire* (*hiw^DN^*; Figure 2C). As with gain-of-function alleles for *faf*, all three loss-of-function alleles for *hiw* also substantially disrupted sleep consolidation at night (Figure 2D). These phenotypes were not observed when expressing wild-type *hiw* (*UAS-hiw^WT^*) with *c929-GAL4* (data not shown).

Sleep loss impairs performance on many cognitive tasks including those that require short-term memory and response-inhibition (Frey et al., 2004; Chuah et al., 2006 Dissel and Shaw, 2013). Thus, to determine whether the reduction in sleep seen in *c929-GAL4>hiw^DN^* flies is the result of a failed ability to generate adequate sleep or the consequence of a reduction in sleep need, we evaluated performance using Aversive Phototaxic Suppression (APS) assay. The APS is an established short-term memory assay that is extremely sensitive to sleep disruption (Seugnet et al., 2008, 2009a,c, 2011a,b; Thimgan et al., 2010, 2015; Dissel et al., 2015a,b,c, 2017). In the APS, flies are individually placed in a T-maze and allowed to choose between a lighted and darkened chamber over 16 trials. Flies that do not display phototaxis during the first block of four trials are excluded from further analysis (Le Bourg and Buecher, 2002; Seugnet et al., 2009a). During 16 trials, flies learn to avoid the lighted chamber that is paired with an aversive stimulus (quinine/humidity). The performance index is calculated as the percentage of times the fly chooses the dark vial during the last four trials of the 16 trial test and short term memory (STM) is defined as selecting the dark vial on two or more occasions during Block 4. Before being tested for STM, flies are first examined to ensure that they exhibit normal photosensitivity and quinine sensitivity (Le Bourg and Buecher, 2002; Seugnet et al., 2008, 2009b). This step is important since changes to sensory thresholds could confound the ability to detect true changes in associate learning (Kahsai and Zars, 2011; Dubnau and Chiang, 2013; Dissel et al., 2015a). As seen in Figure 2E, photosensitivity and quinine sensitivity for *c929-GAL4>UAS-hiw^DN^* flies fall within the range seen in wild-type flies (Seugnet et al., 2008, 2009b) and are not statistically different from both parental controls. Importantly, *c929-GAL4>UAS-hiw^DN^* flies exhibit performance deficits while *c929-GAL4/+* and *UAS-hiw^DN^*/+ parental controls display wild-type STM (Figure 2F). Thus, disrupting the ubiquitin-proteasome system in clock cells interferes with the ability of the flies to obtain needed sleep.

**Figure 3:**
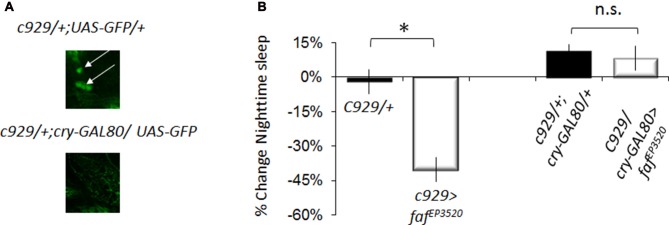
Disrupting *hiw* in clock cells increases waking. **(A)** Anti-GFP immunostaining for *c929-GAL4/+ ;UAS -GFP*/+ and *c929/+ ;UAS-GFP/cryGal80* showing expression in the pars intercerebralis (arrowhead) and the lLN_v_ clock neurons (arrows). **(B)** Blocking expression of *faf* in lLNvs prevents the reduction in night-time sleep (%change *faf^EP3520^*/+; one of two replicates of *n* < 16/group shown; *F*_(3,58)_ = 23.42, *p* = 4.67E-10, *p < 0.05).

The pattern of expression for *pdf-GAL4*, *cry16-GAL4* and *c929-GAL4* all include the large ventral lateral clock neurons (lLNvs) indicating that the effects of *faf* and *hiw* are likely mediated through the large ventral lateral neurons (lLNvs; Grima et al., 2004; Stoleru et al., 2004). However, *c929-GAL4* is expressed in other peptidergic neurons (Park et al., 2008). To determine whether *faf* over-expression in the lLNvs was responsible for the sleep reduction phenotype, we combined *c929-GAL4* with *cry-Gal80*, which targets the GAL4 inhibitor GAL80 to all clock neurons (Stoleru et al., 2004). *cry-GAL80* effectively removed GAL4 mediated induction in the lLNvs as assessed with a *UAS-GFP* reporter (Figure 3A bottom) and abolished the sleep reduction phenotype induced by *c929-GAL4* with *faf^ EP3520^* (Figure 3B). Thus, expression of *faf* in the lLNvs regulates night-time sleep.

**Figure 4:**
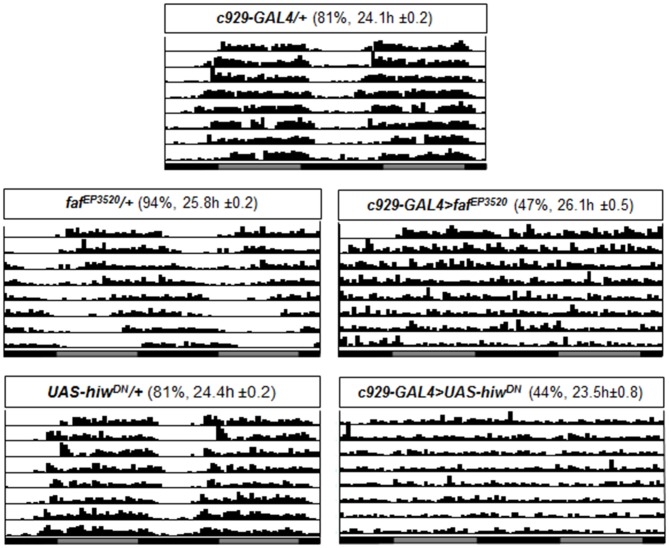
Circadian rhythms are disrupted when *faf^EP3520^* or *UAS-hiw^DN^*, are expressed using c929-GAL4. Representative single fly actograms from DD1 to DD8. Values in parenthesis (%) refer to the percentage of rhythmic flies and the period τ ± SEM. 16–32 flies were evaluated for each group.

Neither *inc*, nor *Cul3* alter free running circadian rhythms when disrupted in clock cells. To determine whether *faf*, and *hiw* would alter free running rhythms, we combined *faf^EP3520^*, and *UAS-Hiw^DN^*, with *c929-GAL4* and *PDF-GAL4*. As seen in Figure 4, >40% of *c929-GAL4 >faf^EP3520^* and *c929-GAL4 >UAS-hiw^DN^* where arrhythmic in free running conditions (Figure 4). Thus, in contrast to *inc* and *Cul3*, expression of *faf* in the lLNvs disrupts circadian locomotor rhythms.

**Figure 5:**
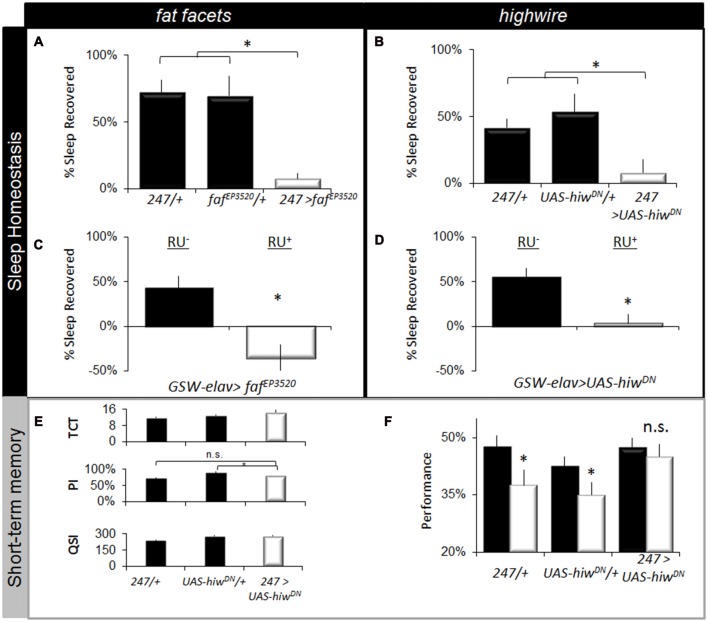
Disrupting *faf^EP3520^* or *hiw* attenuates the negative effects of waking. **(A,B)** Expressing *faf^EP3520^* and *hiw^DN^* in the Mushroom Bodies (MBs) using *247-GAL4* reduces homeostasis. Parental lines are shaded (*n* > 26/group; *F*_(4,195)_ = 18, *p* = 1.41E-12, *p < 0.05, modified Bonferroni correction). **(C,D)** Expressing *faf^EP3520^* and *hiw^DN^* in the adult brain using GeneSwitch (*GSW-elav*) reduced sleep homeostasis (% of sleep recovered in 24 h following a 12 h sleep deprivation (SD); *n* > 20/group). Flies were fed RU486 (RU+) or vehicle (RU−); genotype designations are inset (*t* test, *p < 0.05). **(E)** *247-GAL4 >UAS-hiw^DN^* flies exhibit normal values for the TCT, PSI and QSI that are not statistically different from both *247-GAL4/+* and *UAS-hiw^DN^*/+ parental controls, *p < 0.05 modified Bonferroni test. **(F)** Learning is maintained following SD when *hiw^DN^* is expressed in the MBs (*n* > 8/group, *p < 0.05, modified Bonferroni test).

To determine whether disrupting the ubiquitin-proteasome system would alter other aspects of sleep regulation, we evaluated sleep homeostasis following 12 h of SD. Previous studies have shown that the MB are important for sleep regulation (Joiner et al., 2006; Pitman et al., 2006). Moreover, performance in the APS involves MB neurons which are particularly sensitive to sleep loss (Seugnet et al., 2008, 2009b). As seen in Figures 5A,B, expressing *faf^EP3520^* or *UAS-hiw^DN^* in the MBs using *247-GAL4* significantly attenuated sleep rebound compared to both parental controls. Previous reports suggest that sleep phenotypes are only observed when the ubiquitin-proteasome system is disrupted during development (Pfeiffenberger and Allada, 2012). Thus, we used the inducible GeneSwitch system (Osterwalder et al., 2001) to express *faf^EP3520^* or *UAS-hiw^DN^* in adults. As seen in Figures 5C,D, adult *GSW-elav>faf^EP3520^* and *GSW-elav>UAS-hiw^DN^* flies fed RU486 (RU+) exhibited a significantly reduced sleep rebound compared to genetically identical, vehicle fed siblings (RU−). It should be noted that we and others have consistently reported that RU does not influence a variety of phenotypes including lifespan, sleep, sleep homeostasis, short-term memory, short-term memory following SD, olfactory conditioning, phototaxis, geotaxis, locomotion, the escape response (Mao et al., 2004; Seugnet et al., 2008; Vanderheyden et al., 2013; Dissel et al., 2015c). Thus, disrupting the ubiquitin-proteasome system in adults attenuates sleep homeostasis.

The lack of a homeostatic response following SD may represent either an adaptation that allows animals to better withstand the negative effects of waking, or it may simply indicate that the flies have lost their capacity to respond normally to sleep loss (Seugnet et al., 2011a; Donlea et al., 2012; Dissel et al., 2015b). To distinguish between these two possibilities, we evaluated STM. If flies have reduced sensitivity to sleep loss, they should maintain their ability to learn after SD (Dissel et al., 2015b). However, if they are simply unable to generate the needed compensatory response they should be learning impaired (Dissel et al., 2015b; Dissel and Shaw, 2017). Unfortunately, learning could not be evaluated in *faf* over-expressing flies due to deficits in phototaxis. A previous study has found that expressing *UAS-hiw^DN^* in MBs throughout development does not result in alterations in morphology (Huang et al., 2012). Thus we evaluated the effects of SD on learning in *247-GAL4/UAS-hiw^DN^* flies. As above, we first evaluated photosensitivity and quinine sensitivity to rule out the possibility that changes in behavior could be due to non-associative cues. As seen in Figure 5E, photosensitivity, quinine sensitivity and time to complete the task were similar between genotypes. Importantly, flies expressing *hiw^DN^* in the MB maintain normal learning after SD while the outcrossed parental lines (*247-GAL4*/+ and *UAS-hiw^DN^*/+) are significantly impaired (Figure 5F). The magnitude of the learning deficit observed in the parental lines following SD is similar to that previously reported for sleep deprived wild-type flies and flies lacking MBs (Seugnet et al., 2008). Moreover the deficits in learning following sleep loss in flies are within the range of effect sizes observed following sleep loss in humans and rodents across a number of cognitive domains (Frey et al., 2007; Fu et al., 2007; Pierard et al., 2007). Thus, reducing *hiw* function in the MB confers resistance to SD as measured by both sleep homeostasis and STM.

### Circuit-Dependent Regulation of Sleep by *Rdl*

**Figure 6:**
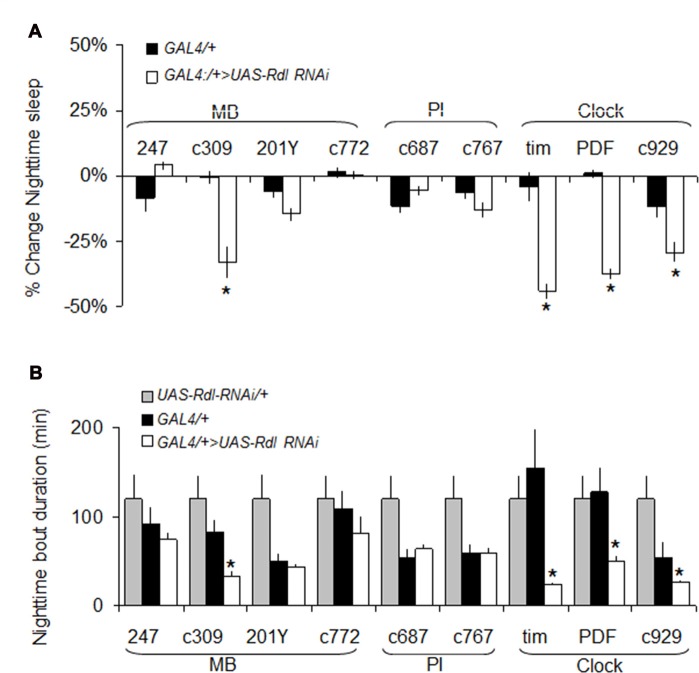
Rdl^RNAi^ screen. **(A)** Changes in nighttime sleep following *UAS-Rdl RNAi* expression using GAL4 drivers that target different areas of the brain involved in sleep regulation (MB, Mushroom Bodies, PI: pars intercerebralis, Clock: clock neurons). Nighttime sleep is presented as % change from *UAS-Rdl RNAi*/+ at age day 8 (*n* > 14/group; *p < 0.05). **(B)** Average nighttime sleep bout duration for the *GAL4>UAS-Rdl RNAi* combinations shown in **(A)** compared to both GAL4/+ and *UAS-Rdl RNAi* /+ (*n* > 14/group, *p < 0.05).

As mentioned above, we have hypothesized that *Rdl* may play a role in insomnia-phenotypes since *Rdl* is downregulated in *ins-l* flies (Seugnet et al., 2009c), is broadly expressed in the brain, including in neurons that impact sleep and waking (Liu et al., 2007; Parisky et al., 2008), and can enhance memory under some circumstances (Liu et al., 2007). While knocking down *Rdl* in lLNvs has been shown to increase waking, its role in other sleep wake-circuits and its impact on sleep homeostasis remains unclear (Agosto et al., 2008; Parisky et al., 2008; Chung et al., 2009). Thus, we conducted a mini-brain screen to express *Rdl^RNAi^* in brain structures associated with sleep regulation. Consistent with previous reports, knocking down *Rdl* in clock neurons reduced night-time sleep and increased sleep fragmentation (Figures 6A,B).

**Figure 7:**
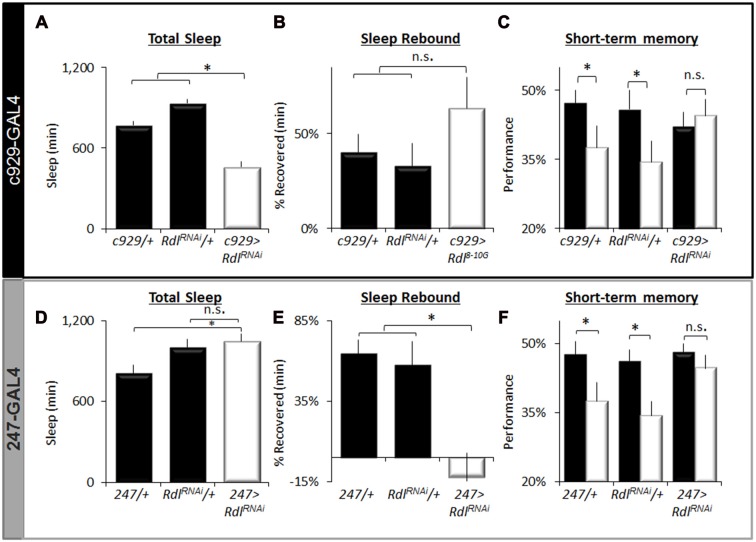
Regulation of sleep by *Rdl*. **(A)** Total Sleep is reduced in *c929-GAL4>UAS-Rdl^RNAi^* flies compared to *c929-GAL4/+* and *UAS-Rdl^RNAi^*/+ parental controls, one-way ANOVA for genotype *F*_(2,68)_ = 39, *p* = 4.86E-12, *p < 0.05, Modified Bonferroni correction. **(B)** Sleep homeostasis in *c929-GAL4>UAS-Rdl^RNAi^* is not statistically different from both *c929-GAL4/+* and *UAS-Rdl^RNAi^*/+ parental controls (*n* > 14/group; *p < 0.05 modified Bonferroni correction). **(C)** Learning under baseline (black) and following a 12 h SD (white) in *c929-GAL4>UAS-Rdl^RNAi^* and *c929-GAL4/+, UAS-Rdl^RNAi^*/+ (*n* > 8/group; main effect for Condition *F*_(1,50)_ = 3.85, *p* = 0.05; *p < 0.05 Modified Bonferroni correction). **(D)** *247-GAL4 <UAS-Rdl^RNAi^* flies exhibit normal levels of Total Sleep that are not statistically different from both *247-GAL4/+* and *UAS-Rdl^RNAi^*/+ parental controls, one-way ANOVA for genotype *F*_(2,40)_ = 4.7, *p* = 0.01, *p < 0.05, modified Bonferroni correction. **(E)** Sleep homeostasis (% of sleep recovered 24 h following a 12 h SD) in *247-GAL4/+*, *UAS-Rdl ^RNAi^*/+ and *247-GAL4>UAS-Rdl^RNAi^* flies (*n* > 14/group; *F*_(2,48)_ = 5.23, *p* = 0.008, *p < 0.05, modified Bonferroni correction). **(F)** Learning under baseline (black) and following a 12 h SD (white) in *247-GAL4/+*, *UAS-Rdl ^RNAi^*/+ and *247-GAL4>UAS-Rdl^RNAi^* (*n* > 8/group; Genotype X Condition (baseline vs. sleep deprived) interaction *F*_(1,83)_ = 3.90, *p* = 0.024; *p < 0.05).

To further characterize the effects of knocking down *Rdl* on sleep homeostasis and STM, we focused on *c929-GAL4* and *247-GAL4*. As seen in Figure 7A, knocking down *Rdl* in *c929-GAL4* expressing cells significantly reduces total sleep time compared *to c929-GAL4/+* and *UAS-Rdl^RNAi^*/+ parental controls as previously described. Similar results were observed with two other RNAi line (*Rdli4-5* and *Rdl^31286^*; data not shown). Although *c929-GAL4>UAS-Rdl^RNAi^* flies are short sleepers, 12 h of additional sleep loss is not accompanied by a larger sleep rebound compared to parental controls (Figure 7B). Importantly, short-sleeping *c929-GAL4>UAS-Rdl^RNAi^* flies exhibit intact STM which is not adversely affected by an additional 12 h of SD (Figure 7C). Note that while performance in *c929-GAL4>UAS-Rdl^RNAi^* flies appears to be slightly lower than parental lines, the difference is not significant and well within the range observed for wild-type flies (Seugnet et al., 2008). Thus, in contrast to the learning impairments seen following disrupting *faf* and *hiw* in lLNvs, knocking down *Rdl* seems to protect flies from the deleterious effects of waking as measured by both a reduced sleep rebound and intact STM.

To determine if knocking down *Rdl* in the MBs would impact sleep homeostasis or STM, we expressed *UAS-Rdl^RNAi^* using *247-GAL4*; knocking down *Rdl* in the MBs throughout development does not result in obvious morphological defects (Liu et al., 2007). As seen in Figure 7D, knocking down *Rdl* in the MBs did not affect total sleep time. However, knocking down *Rdl* in the MBs altered sleep regulation as evidenced by a significantly reduced sleep rebound compared to *247-GAL4/+* and *UAS-Rdl^RNAi^*/+ parental controls (Figure 7E). Although both *247-GAL4/+* and *UAS-Rdl^RNAi^*/+ parental controls displayed impaired STM following SD *247-GAL4>UAS-Rdl^RNAi^* flies maintained normal STM both before and after sleep loss (Figure 7F). No changes were seen in photosensitivity of quinine sensitivity indicating that the observed results were not due to changes in sensory thresholds (data not shown). Together these data suggest that knocking down *Rdl* both in clock neurons (lLNvs) and in the MBs can protect flies from the deleterious effects of SD.

### Differential Modulation of Synaptic Plasticity by *hiw* and *Rdl*

**Figure 8:**
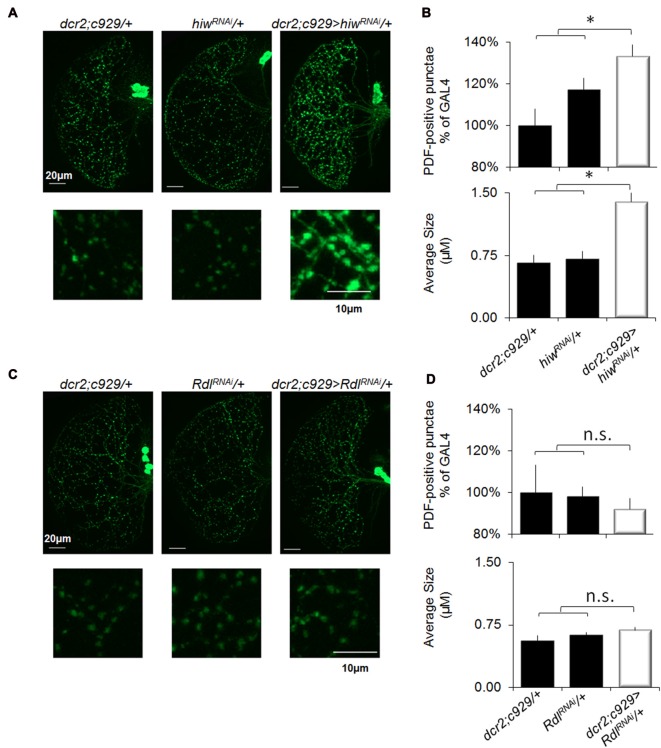
Knocking down *Rdl* in the lLNvs protects flies from sleep-loss induced increases in synaptic markers. **(A)** Representative images of PDF immunohistochemistry of *c929-GAL4/+>UAS-hiw^RNAi^*/+ flies reveals an increase in the number (upper panels) and size (lower panels) of varicosities compared to *c929-GAL4/+* and *UAS-hiw^RNAi^*/+ parental controls. **(B)** Quantification of terminal number expressed as a percentage of GAL4/+ (upper panel) revealed a significant One-way ANOVA for genotype *F*_(2,30)_ = 6.0, *p* = 0.006, *n* = 9–12 each group; *p < 0.05 modified Bonferroni correction. Quantification of bouton size (lower panel) revealed a significant one-way ANOVA for genotype *F*_(2,30)_ = 17.6, *p* = 1.07E-05, *n* = 9–12 each group; *p < 0.05 modified Bonferroni correction. **(C)** Representative images of PDF immunohistochemistry of *c929-GAL4/+>UAS-Rdl^RNAi^*/+ flies does not show any change in the number (upper) or size (lower) of PDF positive punctae compared to *c929-GAL4/+* and *UAS-Rdl^RNAi^*/+ parental controls. **(D)** Quantification of terminal number and size did not reveal a significant one-way ANOVA for number or size *F*_(2,17)_ = 0.2, *p* = 0.79 and *F*_(2,17)_ = 1.9, *p* = 0.18, respectively; *n* = 7 each group.

Previous studies have reported that SD increases synaptic markers (Gilestro et al., 2009; Bushey et al., 2011). Notably, the impact of sleep loss on synaptic plasticity can be readily examined by quantifying pigment dispersing factor (PDF) positive terminals in projections from the lLNvs (Vanderheyden et al., 2013). As noted above, behavioral plasticity is disrupted in short-sleeping *c929/+>UAS-hiw^RNAi^/+* flies but is preserved in short sleeping *c929-GAL4/+>UAS-Rdl^RNAi^*/+ flies (Figures 2A,F, 7A,C). These data lead to the hypothesis that *c929/+>UAS-hiw^RNAi^*/+ flies should exhibit an increase in the number of PDF-positive terminals typical of sleep deprived flies while *c929-GAL4/+>UAS-Rdl^RNAi^*/+ should be unaffected. As seen in Figures 8A–C, short-sleeping *c929/+>UAS-hiw^RNAi^*/+ flies display an increase in the number and size of PDF positive punctae compared to both of their normal sleeping parental controls (*c929/+, UAS-hiw^RNAi^*/+). In contrast, the number, size and intensity of PDF-positive terminals is preserved in short-sleeping *c929-GAL4*/+>UAS-Rdl*^RNAi^*/+ flies compared to their normal sleeping parental controls (*c929-GAL4/+>UAS-Rdl^RNAi^*/+; Figure 8D). Thus, knocking down *Rdl* in lLNvs protects flies from the negative impact of waking as measured both by STM and by examining structural plasticity. In contrast, the increase in synaptic number and morphology induced by knocking down *hiw* in the lLNvs is deleterious to behavioral and structural plasticity.

## Discussion

The de-ubiquitination enzyme *faf* and the E3 ubiquitin ligase *hiw* are evolutionary conserved synaptic proteins that regulate similar targets across phyla (Chen et al., 2000; Burgess et al., 2004; Nakata et al., 2005). The role of these two genes in the context of sleep is unknown. By manipulating *faf* and *hiw*, the present results provide evidence for an important role of specific ubiquitination pathways in the regulation of sleep homeostasis, the vulnerability to sleep loss and the regulation of sleep quotas. Interestingly, the impact of *faf* and *hiw* in conferring vulnerability or resilience to sleep loss is circuit dependent. Previous studies have found that the pan-neuronal disruption of *inc* and *Cul3*, two genes that are also involved in protein degradation pathways, reduces sleep time and attenuates sleep rebound (Stavropoulos and Young, 2011; Pfeiffenberger and Allada, 2012). However, in contrast to *faf* and *hiw*, *inc* and *Cul3* only impact sleep if they are disrupted during development and do not seem to alter sleep when expressed in clock cells. Thus, these data confirm and extend the previous observations by demonstrating that novel ubiquitin pathways can be used in distinct circuits to modulate sleep and the response to sleep loss in adults.

As mentioned above, patients with primary insomnia are able to maintain normal performance on a variety of tasks even though they experience substantial sleep disruption (Riedel and Lichstein, 2000; Orff et al., 2007). These data suggest that insomnia may be a condition where predisposing genetic factors increase the risk for insomnia while simultaneously mitigating against at least some of the deleterious consequences of waking. Although the pan-neuronal expression of *faf^EP3520^* did not increase waking, sleep was substantially disrupted when *faf^EP3520^* was expressed in clock cells. The role of the ubiquitin-proteasome system within clock cells was confirmed by expressing loss-of-function alleles of the E3 ubiquitin ligase *UAS-hiw^DN^*. Importantly, the waking associated with disrupting the ubiquitin-proteasome system within clock cells was associated with STM impairments thereby recapitulating several key features of *ins-l* flies. In addition to disrupting STM, knocking down *hiw* also resulted in quantitative increases in the number and size of PDF-positive punctae similar to that observed during SD. In contrast to clock cells, however, when the ubiquitin-proteasome system was disrupted in the MBs, flies became resistant to SD. Interestingly, a previous report suggests that the ability of *hiw^DN^* to improve memory when expressed in the MBs was not due to its impact on Kenyon Cell morphology (Huang et al., 2012). These data suggest that the activity of a gene in one circuit may disrupt sleep and increase vulnerability to sleep loss while the activity of that same gene in a separate neuronal circuit may protect the animal from the negative consequences of waking. Understanding how *hiw^DN^* protects the MBs during SD will be the focus of future studies.

It is important to emphasize that inducing *faf^EP3520^ and UAS-hiw^DN^* in the adult fly brain using the conditional GeneSwitch *GSW-elav* driver results in a reduction of the homeostatic response following SD. As the induction of the transgene occurred in adult flies, this result indicates that *faf* can impact sleep regulation in the absence of developmental defects. This observation is consistent with the observation that disrupting *hiw* throughout development does not result in obvious structural MB deficits (Huang et al., 2012). Interestingly, knockdown of *inc* and *Cul3* in adults using *GSelav* did not result in short-sleeping flies indicating that *inc* and *Cul3* primarily influence sleep by disrupting neuronal circuitry during development. Indeed, both *inc* and *Cul3* mutants are known to have morphological defects in the MBs (Zhu et al., 2005; Pfeiffenberger and Allada, 2012). Nonetheless, only 20%–30% of *inc* flies were found to have gross morphological defects in their MBs as identified by anti-FASCICLINII (FASII) immunohistochemistry. Since 90% of *inc* flies exhibit sleep disruption, it is possible that disrupted MB morphology may not fully explain the sleep phenotype in *inc* and *Cul3* mutants (Pfeiffenberger and Allada, 2012). While the reduced sleep seen in *inc* flies may not be exclusively due to changes in gross MB morphology, many of the expected morphological defects such as defective re-elaboration of MB dendrites during metamorphosis, and short dendrites that fail to form typical claw-like structure are more readily detected using mosaic analysis (Zhu et al., 2005). Moreover, it is difficult to exclude the possibility that *inc* and *Cul3* may have altered the development of additional neuronal groups that could disrupt circuits which could directly, or indirectly, influence sleep. It is interesting to note that STM remained intact, both during baseline and following SD, in flies expressing *UAS-hiw^DN^* in the MBs indicating that the effects were not due to deficits in MB structure (Seugnet et al., 2008). Indeed, disrupting *hiw* in the adult brain using *GSW-elav* can facilitate long-term memory (Huang et al., 2012). Taken together, these results suggest that disrupting the ubiquitin proteasome system by manipulating *faf* and *hiw* modulates the sensitivity to sleep loss by its effect on the buildup of target proteins, rather than by disrupting neuronal circuits during development.

It is possible that the phenotypes observed after manipulating *faf* and hiw with specific GAL4 drivers could be the result of ectopic expression, unrelated to the endogenous *faf* and *hiw* functions. While this possibility cannot be formally excluded we consider it unlikely for three main reasons: First, if altering *faf* and *hiw* resulted in a non-specific disruption of neuronal function, we would expect to observe sleep phenotype following expression in brain regions previously identified as playing an important role in sleep regulation, such as the MB, the fan shaped body, the pars intercerebralis or dopaminergic neurons. None of these cells types produced a change in sleep time upon *faf* over-expression. In fact only a few *GAL4* drivers resulted in a change of sleep when used to over-express *faf*, and all of these drivers produced a reduction of nighttime sleep. Second, all the phenotypes described here for *faf* over-expression could be phenocopied by a down-regulation of *hiw* function. In the case of *c929-GAL4* expressing cells, this was achieved by both a dominant negative form of *hiw* and RNAi constructs. It is unlikely that these two very different ways of reducing gene activity would result in the same phenotype if they were not specific and directly targeted to endogenous gene function. Finally, the *hiw^DN^* construct specifically blocks the ubiquitin ligase activity of the hiw protein (Wu et al., 2005), suggesting that the phenotypes observed in this study are linked to ubiquitination and not to other aspects of *hiw* function. Indeed, careful studies of temporal requirement for *hiw* function suggest that *hiw* may be maintaining synaptic transmission efficacy throughout the larval life of the animal, independently of its role in morphological plasticity (Wu et al., 2005).

Although it is not yet clear how the ubiquitin-proteasome system could protect flies from the negative effects of waking in specific circuits, a growing body of evidence has emphasized the important role that the ubiquitin system plays in neurons (Kaang and Choi, 2012). As mentioned, both *faf* and *hiw* regulate synaptogenesis and the elimination of synapses at the larval NMJ (DiAntonio et al., 2001). Moreover, the ubiquitin system can be regulated by neuronal activity, and seems to modulate several aspects of presynaptic and postsynaptic neurotransmission. Indeed, ubiquitin pathways not only regulate both excitatory and inhibitory receptors, it appears that the ubiquitin-proteasome system can also impact activity-dependent structural remodeling as evidenced by ubiquitin-dependent changes in spine morphology, size and density (Kaang and Choi, 2012). Interestingly, sleep and waking are also known to modulate activity-dependent changes at the synapse (Maret et al., 2011) suggesting that sleep and waking may rely upon the ubiquitin proteasome system to carry out some of their functions.

Given that, in clock neurons, *faf* and *hiw* increase the vulnerability to waking while in MB neurons *faf* and *hiw* confer resistance to waking, we asked whether modulating the activity of these circuits using an independent molecular pathway would reveal similar outcomes. The expectation that independent molecular pathways can influence insomnia phenotypes seems reasonable given the number and diversity of genes associated with human insomnia (for review see Lind and Gehrman, 2016).

Interestingly, knocking down *Rdl* both in clock cells and in the MB seemed to protect flies from the negative effects of extended waking. In the case of reducing *Rdl* in clock cells, flies maintained their ability to form STM despite being very short sleepers. In fact, short sleeping *c929-GAL4>UAS-Rdl^RNAi^* flies maintained wild-type STM even when they were exposed to an additional 12 h of SD. This latter observation is interesting given that *c929-GAL4/+>UAS-Rdl^RNAi^*/+ flies respond to 12 h of SD with a normal sleep rebound indicating that they have accrued additional sleep debt. The ability to acquire STM during a sleep rebound reinforces previous conclusions that impairments in APS cannot be attributed to increased sleep drive (Seugnet et al., 2008). It should be emphasized that the phenotype observed when *UAS-hiw^DN^* or *UAS-Rdl^RNAi^* are expressed in clock cells looks identical when considering baseline sleep alone. Moreover, one might infer that *c929-GAL4/+>UAS-hiw^DN^*/+ are resistant to sleep loss and that *c929-GAL4/+>UAS-Rdl^RNAi^*/+ are vulnerable to sleep loss based solely upon the absence or presence of a sleep rebound, respectively. However, when one considers STM, a phenotype that is known to be highly responsive to sleep loss in humans, rodents and flies, it becomes clear that the phenotype observed in *c929-GAL4/+>UAS-hiw^DN^*/+ is very different from that observed *c929-GAL4/+>UAS-Rdl^RNAi^*/+ flies. Indeed, *c929-GAL4/+>UAS-hiw^DN^*/+ flies differ not only in behavioral plasticity but also structural plasticity. Identifying the difference in resilience/vulnerability to sleep loss would have been difficult, if not impossible, if we had only used sleep metrics as both the independent and dependent variable as is typically the case (Sehgal and Mignot, 2011; Dissel and Shaw, 2013).

In recent years greater attention has been given to elucidating mechanisms regulating sleep homeostasis (Joiner, 2016). Although genetic evidence has implicated R2 ring neurons of the Ellipsoid body and a subset of neurons in the dorsal Fan Shaped body as integrating signals conveying sleep need (Donlea et al., 2014; Liu et al., 2016; Pimentel et al., 2016), little is known about how or where these signals originate. Our data implicate the MBs as a likely input, into the circuitry underlying sleep homeostasis. It is worth noting that not all manipulations that increase waking are compensated by a subsequent homeostatic response (Joiner, 2016; Allada et al., 2017). For example, sleep rebound is absent or reduced following short periods of starvation or following heat shock during SD (Shaw et al., 2002; Thimgan et al., 2010, 2015; Donlea et al., 2012). Similarly, a recent study has found that activation of acetylcholine neurons induces episodes of waking that are followed by a homeostatic response while wake induced by activating octopaminergic neurons fails to induce a sleep rebound (Seidner et al., 2015). These data suggests the existence of independent arousal circuits that have differential access to homeostatic circuitry (Seidner et al., 2015). However, whereas waking induced by starvation does not disrupt learning and memory (Thimgan et al., 2010), waking induced by the activation of cholinergic and octopaminergic neurons results in learning deficits (Seidner et al., 2015). Thus, while arousal circuits may have differential access to the sleep homeostat, their ability to impair neurons involved in learning and memory may not be as restricted. Indeed, our data indicate that it is also possible for SD to activate homeostatic circuitry while simultaneously protecting against sleep-loss induced cognitive impairments (e.g., sleep deprived *c929-GAL4/+>UAS-Rdl^RNAi^* flies). That is, under some circumstances, sleep homeostasis and cognitive deficits are dissociable. The organization of arousal, homeostatic and cognitive circuits may provide the architecture whereby insomnia-related genes can differentially result in sleep loss and protect flies from the negative effects of waking. Understanding how sleep deprived *c929-GAL4/+>UAS-Rdl^RNAi^* flies can maintain normal cognitive abilities may reveal organizational insights into how extended waking impacts adaptive behavior during insomnia.

## Author Contributions

LS, SD and PJS designed the experiments and analyzed data. LS, SD and MT completed the behavioral and genetic experiments. SD and LC conducted confocal imaging. LS, SD, MT and PJS wrote this manuscript.

## Conflict of Interest Statement

The authors declare that the research was conducted in the absence of any commercial or financial relationships that could be construed as a potential conflict of interest.

## Footnotes

^1^www.Trikinetics.com
